# Massively parallel sequencing uncovered disease‐associated variant spectra of glucose‐6‐phosphate dehydrogenase deficiency, phenylketonuria and galactosemia in Vietnamese pregnant women

**DOI:** 10.1002/mgg3.1959

**Published:** 2022-05-03

**Authors:** Tat‐Thanh Nguyen, Quang‐Thanh Le, Diem‐Tuyet Thi Hoang, Huu Du Nguyen, Thi Minh Thi Ha, My‐Nhi Ba Nguyen, Thanh‐Thuy Thi Ta, Nhat Thang Tran, Thu Huong Nhat Trinh, Kim Phuong Thi Doan, Duc Tam Lam, Son Tra Thi Tran, Thanh Xuan Nguyen, Hong‐Thinh Le, Van Tuan Ha, Manh Hoan Nguyen, Ba‐Liem Kim Le, My Linh Duong, Trung Ha Pham, Anh Tuan Tran, Xuan Lan Thi Phan, Thanh Liem Huynh, Lan‐Phuong Thi Nguyen, Thanh Binh Vo, Duy‐Khang Nguyen Le, Ngoc Nhu Thi Tran, Quynh Nhu Thi Tran, Yen‐Linh Thi Van, Bich‐Ngoc Thi Huynh, Thanh‐Phương Thi Nguyen, Trang Thi Dao, Lan Phuong Thi Nguyen, Truong‐Giang Vo, Thanh‐Thuy Thi Do, Dinh‐Kiet Truong, Hung Sang Tang, Minh‐Duy Phan, Hoai‐Nghia Nguyen, Hoa Giang

**Affiliations:** ^1^ R&D Department Gene Solutions Ho Chi Minh City Vietnam; ^2^ Genetics and Genomics Division Medical Genetics Institute Ho Chi Minh City Vietnam; ^3^ Obstetrics Division Tu Du Hospital Ho Chi Minh City Vietnam; ^4^ Obstetrics and Genetics Department Hung Vuong Hospital Ho Chi Minh City Vietnam; ^5^ Obstetrics Division Can Tho Gynecology Obstetrics Hospital Can Tho Vietnam; ^6^ Medical Genetics Department University of Medicine and Pharmacy, Hue University Hue Vietnam; ^7^ Obstetrics and Gynecology Division Tam Anh Hospital Ho Chi Minh City Vietnam; ^8^ Obstetrics and Gynecology Division Mekong Hospital Ho Chi Minh City Vietnam; ^9^ Obstetrics and Gynecology Department University Medical Center Ho Chi Minh City Vietnam; ^10^ Biomedicine and Genetics Department Hanoi Medical University Hanoi Vietnam; ^11^ Obstetrics and Gynecology Department Can Tho University of Medicine and Pharmacy Can Tho Vietnam; ^12^ Obstetrics and Gynecology Department Vietnam‐Cuba Friendship Dong Hoi Hospital Quang Binh Vietnam; ^13^ Obstetrics and Gynecology Department Hue Central Hospital Hue Vietnam; ^14^ Obstetrics and Gynecology Department Buon Ma Thuot University Hospital Buon Ma Thuot Vietnam; ^15^ Obstetrics and Gynecology Department Dong Nai General Hospital Dong Nai Vietnam; ^16^ Obstetrics Division Sai Gon International Gynecology Obstetrics Hospital Ho Chi Minh City Vietnam; ^17^ Obstetrics and Gynecology Department Long Khanh Hospital Dong Nai Vietnam; ^18^ Center for Molecular and Biomedicine University of Medicine and Pharmacy Ho Chi Minh City Vietnam

**Keywords:** G6PDd, *GAL*, massively parallel sequencing, *PKU*, Vietnam

## Abstract

**Background:**

Several inherited metabolic diseases are underreported in Vietnam, namely glucose‐6‐phosphate dehydrogenase deficiency (G6PDd), phenylketonuria (PKU) and galactosemia (GAL). Whilst massively parallel sequencing (MPS) allows researchers to screen several loci simultaneously for pathogenic variants, no screening programme uses MPS to uncover the variant spectra of these diseases in the Vietnamese population.

**Methods:**

Pregnant women (mean age of 32) from across Vietnam attending routine prenatal health checks agreed to participate and had their blood drawn. MPS was used to detect variants in their *G6PD, PAH* and *GALT* genes.

**Results:**

Of 3259 women screened across Vietnam, 450 (13.8%) carried disease‐associated variants for *G6PD*, *PAH* and *GALT*. The prevalence of carriers was 8.9% (291 of 3259) in *G6PD* and 4.6% (152 of 3259) in *PKU*, whilst *GAL* was low at 0.2% (7 of 3259). Two *GALT* variants, c.593 T > C and c.1034C > A, have rarely been reported.

**Conclusion:**

This study highlights the need for routine carrier screening, where women give blood whilst receiving routine prenatal care, in Vietnam. The use of MPS is suitable for screening multiple variants, allowing for identifying rare pathogenic variants. The data from our study will inform policymakers in constructing cost‐effective genetic metabolic carrier screening programmes.

## Graphical abstract

This study highlights the need for routine carrier screening, where women give blood while receiving routine prenatal care, in Vietnam. The use of massively parallel sequencing is suitable for screening multiple allele variants, allowing for identifying rare disease‐associated variants.NonBreakingSpace; #10;

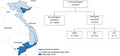

## 1 INTRODUCTION

Glucose‐6‐phosphate dehydrogenase deficiency (G6PDd), an X‐linked hereditary disease, is one of the most common genetic diseases amongst Asian populations (Cappellini & Fiorelli, 2008; Howes et al., 2012). G6PDd affects roughly 400 million people worldwide, mostly in Southern Europe, Africa, the Mediterranean, the Middle East, and Southeast Asia (Bancone et al., 2019; He et al., 2020; Hue et al., 2009; Li et al., 2015; Liu et al., 2019; Louicharoen & Nuchprayoon, 2005; Matsuo et al., 2003; Matsuoka et al., 2004; Monteiro et al., 2014; Nuchprayoon et al., 2002; Ong et al., 2019; Phompradit et al., 2011; Sanephonasa et al., 2021; Satyagraha et al., 2015; Sulistyaningrum et al., 2020; Yusoff et al., 2003; Wang et al., 2008; Zhong et al., 2018).

G6PDd is caused by a deficiency in the enzyme glucose‐6‐phosphate dehydrogenase encoded by the *G6PD* (OMIM: 305900) gene on chromosome Xq28 (Cappellini & Fiorelli, 2008). Clinically, patients with G6PDd present with either the early onset of neonatal hyperbilirubinemia or the late onset of fulminant episodes of hemolysis by specific oxidative agents (such as primaquine and chloroquine, which are prescribed in malarial prophylaxis) or by the intake of fava beans (Cappellini & Fiorelli, 2008; Ong et al., 2017; Tarhani et al., 2021). There is no treatment; thus, the most effective therapy is knowing the disease's presence and preventing exogenously oxidative agents.

Apart from the G6PDd, two other significant genetic metabolic diseases are phenylketonuria (PKU) and galactosemia (GAL). These autosomal recessive diseases are caused by enzyme defects, deficiency, or both, resulting in decreased or abolished metabolism of the amino acid phenylalanine and the sugar galactose, respectively (Hugh‐Jones et al., 1960; Williams et al., 2008). It is estimated that 0.45 million people have phenylketonuria worldwide, with a global prevalence of roughly 1 in 24,000 live births (Hillert et al., 2020). A recent study showed that PKU is amongst Vietnam's seven most common recessive diseases, with a carrier frequency of 2.5% (Tran et al., 2021). The disease frequency of GAL varies from 1 in 23,000 persons in North American and European populations to 1 in 44,000 persons in Asian and African populations (Bosch et al., 2003; Lee et al., 2011; Ruiz et al., 1999; Senemar et al., 2011).

Certain variations in the phenylalanine hydroxylase (*PAH* [OMIM: 612349]) gene cause PKU. The major clinical manifestations of PKU are growth failure, hypopigmentation, microcephaly, seizures, and mental retardation (Al Hafid & Christodoulou, 2015; Williams et al., 2008). Untreated PKU results in intractable seizures, irreversible intellectual impairment, and motor dysfunctions. Two drugs that target phenylalanine metabolism are on the market. Still, an urgent need remains to diagnose the disease early to prevent the irreversible morbidity associated with the disease. Restricted diet, beginning as early as possible, is still the key treatment. (Mahan et al., 2019; Vockley et al., 2014).

Lastly, classic galactosemia (type 1) is caused by variants in the *GALT* (OMIM: 606999) gene located on chromosome 9p13 and is the most common and severe form of the condition (Fridovich‐Keil et al., 2011; Kotb et al., 2019; Wada et al., 2018). Babies with GAL may present a few days after birth with jaundice, hepatomegaly, progressive liver cirrhosis, bleeding or sepsis. Most importantly, the disease can be life‐threatening if not diagnosed accurately or treated appropriately (Bosch et al., 2003; Ruiz et al., 1999).

These three conditions are amongst the five most critical conditions being screened for in the national newborn screening programme. The newborn screening programme has not been implemented effectively yet, <30% of newborns are screened, and the programme is only available in big cities. Therefore, the Ministry of Health sets the target that by 2030 at least 70% of pregnant women need to be screened for three critical diseases and 90% of newborns need to be screened for the five commonest diseases. The intervention, along with newborn screening programme, will help to achieve the target. According to ACOG—Committee Opinion number 690, prenatal carrier screening does not replace the newborn screening, nor does newborn screening diminish the potential benefit of prenatal carrier screening.

The advent of massively parallel sequencing (MPS) allows the simultaneous detection of multiple genetic variants, including novel variants in numerous genes. Thus, these genetic diseases can be accurately detected using MPS within a single test, substantially reducing the testing cost. Knowing the disease‐associated variant spectra of recessive hereditary diseases in a particular population can aid the design of diagnosis, disease‐severity prediction and proactive intervention. However, these data are limited by geographic variations and ethnic‐specific differences (Hillert et al., 2020; Howes et al., 2012; Senemar et al., 2011). Despite previously reported studies, there is still a lack of large‐scale prenatal genetic screening studies about these three common metabolic diseases amongst Vietnamese and other Asian populations (Lee et al., 2011; Matsuo et al., 2003; Senemar et al., 2011; Tarhani et al., 2021; Tran et al., 2021). In parallel with the benefits of a newborn genetic screening programme, a prenatal screening programme for pregnant women will bring more benefits in providing carriers with their genetic data. Currently, efforts are focused on the women, as there is a higher likelihood of agreeing to the testing by them at a routine prenatal visit. As a result, paternal and maternal variant carriers will be offered comprehensive genetic counselling programmes so they can be proactive in selecting the most appropriate preemptive or palliative treatments for their child and can adequately plan future pregnancies.

To fill in knowledge gaps and further inform policies in designing prenatal screening programmes cost‐effectively, we conducted a large‐scale study using MPS to screen 3259 Vietnamese pregnant women in outpatient clinic settings to determine the disease frequencies and disease‐associated variant spectra of these genetic diseases.

## 2 METHODS

### 2.1 Study sites and participants

**FIGURE 1:**
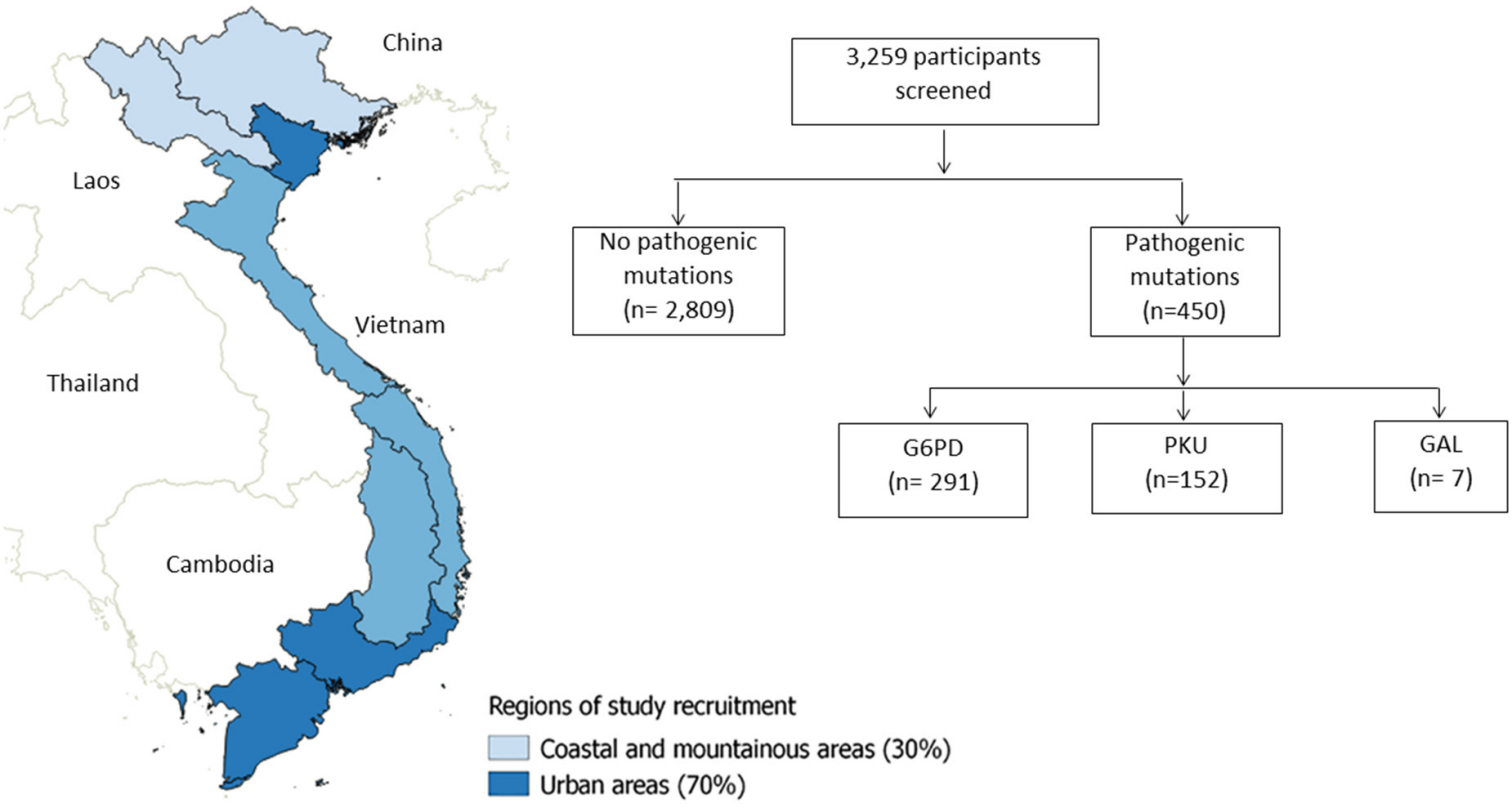
Map of regions in Vietnam where participants were recruited (left side) and the study screening flowchart (right side). The densely‐populated urban areas (dark blue) contributed roughly 70% of the samples, whilst the remaining less densely populated coastal and mountainous provinces (light blue) contributed 30% of the studied samples. G6PDd, Glucose‐6‐phosphate dehydrogenase deficiency; PKU, Phenylketonuria; GAL, Galactosemia

A large‐scale, multicenter, cross‐sectional descriptive study was conducted across Vietnam from the beginning of June to the end of August 2020 **(**Figure 1 **)**. The Vietnamese pregnant women visiting obstetric clinics and hospitals for their routine health checks were screened and invited to participate in this study.

### 2.2 Sample collection

Maternal venous blood samples were taken and stored in blood cell collection tubes (Streck) according to the manufacturer's instructions. Genomic DNA was extracted from maternal buffy coat using the MagMAX™ DNA Multi‐Sample Ultra 2.0 Kit on the Kingfisher Flex System (Thermo Fisher) following the manufacturer's protocol. The blood samples were anonymized, and researchers had access to only de‐identified samples.

### 2.3 Library preparation and targeted sequencing

Sequencing libraries were prepared from genomic DNA using NEBNext® Ultra™ II FS DNA Library Prep Kit (New England Biolabs) according to the manufacturer's instructions. DNA concentration was quantified using QuantiFlour® dsDNA kit (Promega). Equal amounts of 150 ng per sample library were pooled together and hybridized with xGen Lockdown Probes, including *G6PD*, *PAH*, and *GALT* (Table 1) genes (IDTDNA). Sequencing was performed using paired‐end 2x75bp reagent kits on NextSeq™ 550 system (Illumina). The minimum coverage depth in the target regions for all samples is 100X, with a minimum 95% base higher than 20X.

**TABLE 1:** GenBank reference sequence and accession number for genes studied

Gene name	Gene symbol	Chromosome	NCBI Reference Sequence
Homo sapiens glucose‐6‐phosphate dehydrogenase	*G6PD*	X	NG_009015.2
Homo sapiens phenylalanine hydroxylase	*PAH*	12	NG_008690.2
Homo sapiens galactose‐1‐phosphate uridylyltransferase	*GALT*	9	NG_009029.2

### 2.4 Variant calling

Samples were de‐multiplexed using the dual‐indexed sequences. Sequencing quality was assessed with the FastQC package (version 0.11.9) (Babraham Bioinformatics, 2021). Using Burrows‐Wheeler Aligner software, the paired‐end reads were aligned to the human reference genome (build GRCh38) (Li, 2013). Sequence reads were used to call variants with the GATK 3.8 package after removing duplication using MarkDuplicates from Picard tools (Van der Auwera et al., 2013). All variants were annotated using the Ensemble Variant Effect Predictor programme with reference to dbSNP (version 151) and the ClinVar database (Landrum et al., 2013; McLaren et al., 2016; Sherry et al., 2001).

The sequencing results were aligned to reference genome GRCh38/hg19 to identify variants. They were classified based on the ClinVar database from the US National Institutes of Health. If they were not in the Clinvar database, they were classified according to the American College of Medical genetics guidelines (Rehm et al., 2013). Variants were divided into three classes: (i) Pathogenic and likely pathogenic (Pathogenic): a variant that shows adequate scientific evidence relating to the disease; (ii) Variants of Uncertain Significance (VUS): a variant that is insufficient in incidence or conflicting of disease‐association; (iii) Benign and likely benign (Benign): a variant that has enough scientific evidence not to increase disease incidence. Only disease‐associated variants related to clinical symptoms were reported.

### 2.5 Statistical analysis

Descriptive statistics were used to determine the frequency and proportions of pathogenic variants. Stata statistical software version 16.0 was used for the data analysis.

## 3 RESULTS

A total of 3259 pregnant Vietnamese women were screened for variants in *G6PD*, *PKU*, and *GAL*. The mean age of participants was 32 years old, with a standard deviation of 5 years. The combined carrier frequency for *G6PD, PKU*, and *GAL* was 450 of 3259 (13.8%) amongst studied pregnant women (Figure 1).

### 3.1 Identification of *G6PD* disease‐associated variants

Amongst the 3259 participants, 291 (8.9%) harboured *G6PD* disease‐associated variants **(**Table 2 **).** All the variants were missense variants. Five disease‐associated variants associated with G6PDd phenotypes were identified, including c.961G>A (p.Val321Met), c.1466G>T (p.Arg489Leu), c.1478G>A (p.Arg493His), c.1360C>T (p.Arg454Cys) and c.653C>T (p.Ser218Phe). Notably, the most prevalent *G6PD* disease‐associated variant was Viangchan/Jammu variant, c.961G>A (p.Val321Met) with a carrier frequency of 2.03%, whilst the rarest variant was the Sassari variant c.653C>T (p.Ser218Phe). Interestingly, there was one participant harbouring compound Jammu and Union variants.

**TABLE 2:** Prevalence of pathogenic G6PD variants amongst Vietnamese women (N = 3259)

Variant names	Nucleotide substitutions	Amino acid substitutions	Heterozygous, N (%)	Homozygous, N (%)	Total, N (%)
Viangchan‐Jammu	NM_000402.4 (G6PD): c.961G>A	Val321Met	130 (3.99)	1 (0.03)	131 (4.02)
Taiwan‐Hakka	NM_000402.4 (G6PD): c.1466G>T	Arg489Leu	77 (2.36)	‐	77 (2.36)
Anant	NM_000402.4 (G6PD): c.1478G>A	Arg493His	44 (1.35)	‐	44 (1.35)
Union	NM_001360016.2 (G6PD): c.1360C>T	Arg454Cys	38 (1.17)	‐	38 (1.17)
Sassari	NM_000402.4 (G6PD): c.653C>T	Ser218Phe	1 (0.03)	‐	1 (0.03)
Total			290 (8.92)	1 (0.03)	291 (8.93)

*Note*: One study participant carried dual mutations of the Jammu and Union variant; − indicates not found.

The carrier frequencies of the five *G6PD* disease‐associated variant types in this study are compared to other Asian populations in Table 3. The second most common variant is Taiwan/Hakka, with a carrier frequency of 1.18%, followed by the Anant and Union variants. However, the Sassari was the least observed variant amongst Vietnamese women, with a carrier frequency of 0.02% (Table 3).

**TABLE 3:** Variant frequency (%) of G6PD variants amongst Vietnamese women in our study compared with other Asian populations

Variant	Vietnam^a^	Thailand	Myanmar	China	Cambodia	Laos
Viangchan‐Jammu	2.03	2.30–5.70^b^	<1.0^b^	0.03^c^ ^,^ ^d^	8.20–24.80^b^	4.80–8.90^b^
Taiwan‐Hakka	1.18	–	–	–	–	–
Anant	0.68	–	–	–	–	–
Union	0.58	0.20^e^	–	0^c^, 0.08^f^ ^,^ ^g^	<1.00	<1.00
Sassari	0.02	–	–	–	–	–

*Note*: Dashes denote the identified variants are not found in other Asian populations.

^a^ Data from this study.

^b^ Bancone et al. (2019).

^c^ Li et al. (2015).

^d^ Kachin Jingpo Ethnic group.

^e^ Phompradit et al. (2011).

^f^ Hakka ethnic group.

^g^ Zhong et al. (2018).

### 3.2 Identification of phenylketonuria disease‐associated variants

Regarding phenylketonuria, 152 (4.66%) pregnant women carried disease‐associated variants in the *PAH* gene **(**Table 4 **)**. Seventeen pathogenic variants associated with the PKU phenotype were identified. The most predominant variant was c.516G>T(p.Gln172His) with a carrier frequency of 1.83% amongst Vietnamese women, followed by c.1223G>A (p.Arg408Gln) with a carrier frequency of 0.14%, whilst the remaining variants had frequencies ranging from 0.015% to 0.003%. The types of mutations in *PAH* comprised 93.4% missense, 4% stop‐gained, 1.3% inframe deletion and 1.3% frameshift deletion.

**TABLE 4:** Prevalence of pathogenic phenylketonuria variants amongst Vietnamese women (N = 3259)

Variants	Nucleotide substitutions	Amino acid substitutions	Heterozygous, N (%)	Variant frequency, %
1	NM_000277.3(PAH): c.516G>T	Gln172His	119 (3.65)	1.83
2	NM_000277.3(PAH): c.1223G>A	Arg408Gln	9 (0.28)	0.14
3	NM_000277.3(PAH): c.940C>A	Pro314Thr	3 (0.09)	0.05
4	NM_000277.3(PAH): c.618C>A	Tyr206Ter	3 (0.09)	0.05
5	NM_000277.3(PAH): c.960G>C	Lys320Asn	2 (0.06)	0.03
6	NM_001354304.2: c.1174T>A	Phe392Ile	2 (0.06)	0.03
7	NM_000277.3(PAH): c.722del	Arg241fs	2 (0.06)	0.03
8	NM_000277.3(PAH): c.728G>A	Arg243Gln	2 (0.06)	0.03
9	NM_000277.3(PAH): c.331C>T	Arg111Ter	2 (0.06)	0.03
10	NM_000277.3(PAH): c.721C>T	Arg241Cys	1 (0.03)	0.015
11	NM_000277.3(PAH): c.43_44CT	Leu15_Ser16insTer	1 (0.03)	0.015
12	NM_000277.3(PAH): c.439C>T	Pro147Ser	1 (0.03)	0.015
13	NM_000277.3(PAH): c.472C>T	Arg158Trp	1 (0.03)	0.015
14	NM_000277.3(PAH): c.510T>A	His170Gln	1 (0.03)	0.015
15	NM_000277.3(PAH): c.208_210del	Ser70 del	1 (0.03)	0.015
16	NM_000277.3(PAH): c.281_283TCA	Ile95 del	1 (0.03)	0.015
17	NM_000277.3(PAH): c.1162G>A	Val388Met	1 (0.03)	0.015
Total			152 (4.66)	2.33

*Note*: No homozygous mutations of the *PAH* gene were detected.

### 3.3 Identification of galactosemia disease‐associated variants

The *GALT* variant was scarce amongst Vietnamese women, with only seven (0.21%) participants harbouring pathogenic variants in the *GALT* gene (Table 5). All the mutations were missense variants. Four disease‐associated variants associated with GAL phenotypes were identified, including c.593T>C (p.Ile198Thr), c.1034C>A (p.Ala345Asp), c.602G>A (p.Arg201His) and c.691C>T (p.Arg231Cys). The overall carrier frequency of all *GALT* variants was approximately 0.11% amongst Vietnamese women.

**TABLE 5:** Prevalence of pathogenic galactosemia variants amongst Vietnamese women (N = 3259)

Variants	Nucleotide substitutions	Amino acid substitutions	Heterozygous, N (%)	Variant frequency, %
1	NM_000155.4(GALT): c.593T>C	Ile198Thr	4 (0.12)	0.061
2	NM_000155.4(GALT): c.1034C>A	Ala345Asp	1 (0.03)	0.015
3	NM_000155.4(GALT): c.602G>A	Arg201His	1 (0.03)	0.015
4	NM_000155.4(GALT): c.691C>T	Arg231Cys	1 (0.03)	0.015
Total			7 (0.21)	0.11

*Note*: No homozygous mutations of the *GALT* gene were detected.

## 4 DISCUSSION

This study aimed to determine the prevalence of carriers of three common hereditary genetic diseases amongst Vietnamese pregnant women, regarded as potential carriers of disease‐associated variants to newborns. We comprehensively characterized the disease‐associated variant spectra of these diseases to fill in the genetic knowledge gaps and further inform prenatal screening programmes and policies.

First, G6PDd, one of the most common enzymatic disorders, involves the pentose phosphate pathway of human erythrocyte metabolism. The global prevalence of G6PDd disease ranges from roughly 2% up to 80%, mainly depending on the geographic variations and ethnicities (Bancone et al., 2019; He et al., 2020; Hue et al., 2009; Li et al., 2015; Liu et al., 2019; Louicharoen & Nuchprayoon, 2005; Matsuo et al., 2003; Matsuoka et al., 2004; Monteiro et al., 2014; Nuchprayoon et al., 2002; Ong et al., 2019; Phompradit et al., 2011; Sanephonasa et al., 2021; Satyagraha et al., 2015; Sulistyaningrum et al., 2020; Wang et al., 2008; Yusoff et al., 2003; Zhong et al., 2018). This wide distribution highlights the need for a G6PDd screening programme to detect potential maternal carriers harbouring unexpressed *G6PD* variants and prevent disease complications. This study may be the first large‐scale, multicenter, cross‐sectional screening study in Vietnam. Due to the X‐linked nature of this disease, males are not considered carriers. This study showed the prevalence of *G6PD* carriers to be 8.9% amongst Vietnamese pregnant women, close to the previously reported prevalence of roughly 10% amongst the healthy populations in some Latin American and Southeast Asian countries (Matsuoka et al., 2004; Monteiro et al., 2014; Nuchprayoon et al., 2002; Phompradit et al., 2011; Sanephonasa et al., 2021). Of the five disease‐associated *G6PD* variants detected amongst 290 unrelated variant‐harbouring participants in this study, Viangchan/Jammu and Union are still the most frequently seen variants in Vietnam as well as other Southeast Asian populations with relatively high carrier frequencies amongst the female population (Hue et al., 2009; Louicharoen & Nuchprayoon, 2005; Nuchprayoon et al., 2002; Ong et al., 2019; Phompradit et al., 2011; Satyagraha et al., 2015; Yusoff et al., 2003).

Most importantly, apart from Viangchan/Jammu and Union variants, prevailing *G6PD* variants in Chinese, Thai, and other Southeast Asian populations were not found in this study (He et al., 2020; Li et al., 2015; Liu et al., 2019; Louicharoen & Nuchprayoon, 2005; Matsuoka et al., 2004; Nuchprayoon et al., 2002; Ong et al., 2019; Phompradit et al., 2011; Sanephonasa et al., 2021; Satyagraha et al., 2015; Sulistyaningrum et al., 2020; Wang et al., 2008; Yusoff et al., 2003; Zhong et al., 2018). On the contrary, the remaining three pathogenic G6PD variants in this study, including Taiwan/Hakka, Anant and Sassari variants (which are very rarely observed in Southeast Asia and China), significantly add to the archive of previously reported Vietnamese *G6PD* disease‐associated variants, including Vietnam 1, G7 > A (c.7G>A, p.Glu3Lys) and Vietnam 2, T10148 > G (c.117T>G, p.Phe66Cys) (Hue et al., 2009).

Second, there is no published comprehensive study of *PAH* disease‐associated variants in Vietnam, and this is the first study to characterize the genetic features associated with PKU amongst pregnant women in Vietnam. Our study showed that 4.6% of Vietnamese pregnant women identified as *PAH* carriers were asymptomatic at study enrollments. Our study figure was higher than the previously reported *PAH* frequency of 2.5% (or 1 in 40 individuals) in a cohort of 985 participants from the general population in Vietnam (Tran et al., 2021). This earlier publication included 985 participants spanning several age groups (fetuses, neonates, and adults) and both sexes (46% female and 54% male), whilst this study had 3259 pregnant women. This difference in cohorts may explain the difference. PKU is mostly observed in Southern European or Hispanic populations with a carrier frequency of 0.7%, whilst the disease is rarely seen in Eastern Asian populations (Lazarin et al., 2012). Over 1000 *PAH* variants have been reported, corresponding to various clinical PKU phenotypes from asymptomatic to mild to severe presentations (Hillert et al., 2020; Lin et al., 1992; Liu et al., 2015). The most prevalent *PAH* variants were c.1222C>T (p.Arg408Trp), c.1066‐11G>A and c.782G>A (p.Arg261Gln) (Hillert et al., 2020). The c.516G>T (p.Gln172His) was predominant in this study, with the highest variant frequency of 1.83% in Vietnamese women carriers. Otherwise, this variant was reported in the prenatal screening of the Chinese fetal population with sparse frequency (Liu et al., 2015). Furthermore, the second most frequent *PAH* variant in this study was c.1223G>A (p.Arg408Gln), also seen in Chinese, Taiwanese, and Japanese populations (Hillert et al., 2020; Liang et al., 2014; Lin et al., 1992; Okano et al., 1998). In addition, the remaining *PAH* variants with very low carrier frequency in this study were observed in Eastern Asian countries, except for c.722del (p.Arg241fs), c.43_44CT (p.Leu15_Ser16insTer), and c.439C>T (p.Pro147Ser) in European regions (Landrum et al., 2013). However, all *PAH* variants in our study were heterozygous, and this finding is consistent with a previously reported heterozygous predominance (Blau et al., 2014; Hillert et al., 2020; Zschocke et al., 1995). The results from our study reveal that PKU carrier frequency was higher amongst the Vietnamese and other Eastern Asian populations, and it appears that PKU is underreported (Lazarin et al., 2012; Tran et al., 2021).

Third, classic GAL is a potentially fatal autosomal recessive inborn error of metabolism, affecting more American, Australian and European populations than Asian people (Bosch et al., 2003; Lee et al., 2011; Ruiz, et al., 1999; Senemar et al., 2011). An estimated 1% of the North American population are carriers, corresponding to a disease frequency of 1 in 40,000 people (Bosch et al., 2003). Our study showed that the prevalence of GAL amongst pregnant carriers was 0.2% (2 in 1000 pregnant women), which was higher than 5 in 24,000 neonates reported in southern Iran (Senemar et al., 2011). Approximately 336 different variants of GAL were reported, and the majority of these originated from the pathogenic missense variants, significantly reducing enzyme activity or an enzyme deficiency (Calderon et al., 2007). The most frequently observed variants reported were recognized disease‐associated variants, including c.855G>T (p.Lys285Asn), c.584T>C (p.Leu195Pro), c.626A>G (p.Tyr209Cys) and c.512T>C (p.Phe171Ser), c.404C>T (p.Ser135Leu) and c.940A>G (p.Asn14Asp) (Berry, 2000). However, none of these GAL‐associated variants was found in this study. Another noteworthy point was that two *GALT* variants in our study, c.593T>C (p.Ile198Thr) and c.1034C>A (p.Ala345Asp), were both very rarely seen elsewhere.

Most significantly, compared to the newborn screening programme for G6PDd, PKU and GAL, the prenatal screening for these diseases will provide pregnant women and their partners with a thorough understanding of the variants they carry. The physicians will also counsel the fathers to get tested, so the couple has a clear understanding of the genetic possibilities of their offspring. Knowledge of their genetic makeup will allow the couple to be more proactive in selecting the appropriate preemptive or palliative treatments as well as obtain genetic counselling for future pregnancy plans. As a result, this should substantially reduce the disease burden and improve the quality of life of those carrying the variants. In this study, MPS demonstrated its advantage over the conventional gene sequencing technique. The MPS can investigate multi‐variants in many regions of various genes. Therefore, the MPS will expand a wide array of genes explored and enhance productivity and cost‐effectiveness. As a result, this will make it feasible for broad applicability in population genetic screening programmes.

Our study has one major limitation. Sampling bias is considered inherent to the cross‐sectional study design. This study recruited participants from multiple centres across Vietnam to obtain a well‐represented sampling of the Vietnamese population. However, since ethnicity data were not collected, we could not present the disease prevalence at the level of each ethnic group in Vietnam. Therefore, we did not capture the possible differences amongst various Vietnamese ethnic groups.

## 5 CONCLUSION

This study highlights the practical need for prenatal screening of genetic and metabolic diseases by MPS. This large‐scale study characterized the prevalence and expanded the disease‐associated variant spectra of *G6PD, PKU* and *GAL* amongst pregnant women, highlighting differences in the Vietnamese population compared to other people. Our results will help policymakers and medical experts devise appropriate strategies for prenatal diagnostic programmes and counselling plans for genetic diseases.

## CONFLICT OF INTEREST

We declare that there is no conflict of interest.

## ETHICS STATEMENT

This study was approved by the ethics and scientific committee of the University of Medicine and Pharmacy, Ho Chi Minh City, Vietnam. The study complied with the guidelines set by the University of Medicine and Pharmacy, Ho Chi Minh City, in handling human genetic data of all participants. All study participants completely understood the study objectives and gave their written informed consent.

## AUTHOR CONTRIBUTIONS

*Study concept and design*: Duy PM, Nghia NH, Hoa G, Sang TH; *Obtaining Funding*: Thanh NT; *Data acquisition*: Thanh NT, QTL, DTTH, HDN, TMTH, MNBN, TTTT, NTT, THNT, KPTD, DTL, STTT, TXN, HTL, VTH, MHN, BLKL, MLD, THP, ATT, XLTP, TLH, LPTN; *Laboratory work*: Thanh NT, TBV, DKNL, NNTT, QNTT, YLTV, BNTH, TPTN, TTD, LPTN, TGV, TTTD, DKT; *Data analysis*: Thanh NT, Hoa G. *Drafting the manuscript*: Thanh NT; *Critical revision of the manuscript for intellectual content*: Duy PM, Nghia NH, Hoa G, Sang TH. All authors contributed to and approved the final manuscript.

## Data Availability

Data available on request due to ethical restrictions.
